# Altered behavioral and electrophysiological responses to social fairness in manic and euthymic patients with bipolar disorder

**DOI:** 10.1002/brb3.2289

**Published:** 2021-07-21

**Authors:** Vin Ryu, Ra Yeon Ha, Hyun‐Sang Cho

**Affiliations:** ^1^ Department of Psychiatry National Center for Mental Health Ministry of Health and Welfare Seoul South Korea; ^2^ Department of Psychiatry Yong In Mental Hospital Gyeonggi South Korea; ^3^ Department of Psychiatry College of Medicine Yonsei University Seoul South Korea; ^4^ Institute of Behavioral Science in Medicine College of Medicine Yonsei University Seoul South Korea

**Keywords:** bipolar disorder, feedback‐related negativity, rejection rate, ultimatum game

## Abstract

**Background:**

Individuals with bipolar disorder show mood instability, including heightened anger and impulsivity. The Ultimatum Game (UG) is a tool used to evaluate emotional and social decision‐making strategies. We investigated behavioral and electrophysiological responses to subjectively fair or unfair offers in the UG in patients with bipolar I disorder.

**Methods:**

Twenty‐four manic patients, 20 euthymic patients, and 30 healthy controls participated in this study. We analyzed their behaviors and collected electroencephalography data with which to analyze feedback‐related negativity (FRN) as they played in the UG as responders.

**Results:**

Manic patients exhibited significantly higher rejection rates for unfair offers than euthymic patients and healthy controls. Healthy individuals exhibited a greater (i.e., more negative) FRN amplitude in response to unfair offers than to fair offers, whereas euthymic patients exhibited a greater FRN amplitude in response to fair offers compared with unfair offers. Manic patients exhibited no difference in FRN amplitudes between fair and unfair offers.

**Conclusions:**

The current data suggest that different behavioral responses and FRN amplitude patterns can be associated with characteristic manifestations of mood instability in manic bipolar patients. In addition, electrophysiological alterations in response to unfair offers may be a trait abnormality independent of mood state.

## Graphical abstract

We found FRN response changes with higher rejection rates during ultimatum game in bipolar patients.

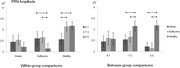

## 1 INTRODUCTION

Bipolar disorder is characterized by mood instability, anger, and aggressive behavior (Bonsall et al., 2012; Perroud et al., 2008 ). The prevalence of violent behavior in patients with bipolar disorder is approximately 25%, compared to less than 1% in the general population (Latalova, 2009). Aggression based on anger is an important characteristic of patients with bipolar disorder (Ballester et al., 2014). This characteristic is related to symptom severity, but is relatively high even during stable mood states. Furthermore, heightened anger has been reported as a subsyndromal symptom of bipolar disorder (S. J. Dutra et al., 2014).

Individuals face a conflict between rational thinking and emotional arousal in response to social fairness (Fehr & Fischbacher, 2003; Frith & Frith, 2008; Hewig et al., 2011 ). Emotional decision‐making is processed in the prefrontal cortex, amygdala, limbic system, and anterior cingulate cortex (ACC). Patients with bipolar disorder and healthy controls perform similarly on probabilistic classification tasks, which evaluate learning ability. However, the Ultimatum Game (UG), which evaluates emotional responses and punishment (Koenigs & Tranel, 2007), reveals an imbalance between reward and punishment learning as well as residual anger, in patients with bipolar disorder (Duek et al., 2014).

The UG, which is also called the “take it or leave it game” (Nelissen et al., 2009), is based on the game theory (Güth et al., 1982). In the UG, one participant plays the role of the “proposer,” and another participant plays the role of the “responder.” The proposer is given a sum of money and is instructed to offer part of this sum to the responder. The game has two outcomes. One outcome is that the responder accepts the offer and receives his or her share. This decision is rational because neither participant receives anything if the offer is rejected. The other outcome is that the responder rejects the offer. In this case, neither the responder nor the proposer receives anything. Thus, if the responder feels that the offer is unfair, he or she can take revenge by making a personal sacrifice. This decision is fundamentally emotional (Falk & Fischbacher, 2006) and is associated with anger, aggressive behavior, and low serotonin concentration (Crockett et al., 2010; Mehta & Beer, 2010; Pillutla & Murnighan, 1996; Sanfey et al., 2003). The UG measures the level of altruistic punishment. By rejecting the unfair offer, participants can punish the unfair offer with “costly” or “altruistic” punishment. The “cost” of punishment means the potential earning that responders would otherwise receive. Altruistic punishment is an impulsive act driven primarily by an emotional reaction to unfairness (Koenigs & Tranel, 2007; Sanfey et al., 2003; Tabibn, 2008).

The social utility theory which focuses on guilt from getting more than others, explain the rejection of unfair offers, which predicts rejection of low ultimatum offers to reduce envy and guilt (Camerer, 2003). Another explanation that focuses on the human instinct to reciprocate, is that punishing others for unfair offers to keep up social status reputation persist even while playing game (Nowak et al., 2000).

Feedback‐related negativity (FRN) is a negative deflection in the event‐related potential (ERP), the maximum amplitude of which is recorded at the scalp over the frontal brain region (especially electrode Fz) (Van den Berg et al., 2011) approximately 250–300 ms after the onset of a negative event, such as negative performance feedback compared to positive performance feedback (Holroyd & Coles, 2002; Miltner et al., 1997) or a gambling loss compared to a win (Hewig et al., 2011; Yeung & Sanfey, 2004). Negative amplitudes have been observed in response to losses in which punishment is worse than expected. Punishment leads to negative affective responses and is linked to the concept of habitual or trait‐like differences in negative affect (Jeffrey A Gray, 1994; Jeffrey Alan Gray & McNaughton, 2003). In the UG, greater FRN amplitudes to unfair offers reflect negative responses and may lead to an increased likelihood of remedial action in terms of rejection of unfair offers (Qu et al., 2013).

In this study, we assessed decision‐making in patients with bipolar I disorder as they played the UG. Specifically, we assessed whether patients with bipolar disorder would show more altruistic behavior in response to unfair offers than healthy controls. We also evaluated whether altered decision‐making processes were observed in euthymic patients. We hypothesized that altered emotional decision‐making processes in patients with bipolar disorder may lead to high rejection rates and a related increase in FRN. If so, we anticipated that this alteration would also be observed in euthymic patients.

## 2 METHODS

### 2.1 Participants

Forty‐four patients with bipolar I disorder (24 manic, 20 euthymic) were recruited from inpatients and outpatients at the Severance Mental Health Hospital of Yonsei University Health System. A diagnosis of bipolar disorder was made according to the Diagnostic and Statistical Manual of Mental Disorders, 4th edition (DSM IV) criteria by two psychiatrists (R.Y.H. and H.S.C.) using the Mini‐International Neuropsychiatric Interview (MINI) (Sheehan et al., 1997). Patients with other psychiatric illnesses, such as schizoaffective disorder, personality disorder, comorbid substance abuse or dependence, rapid‐cycling bipolar disorder, history of closed head injury, mental retardation, neurological disorder, or any other current axis I disorder, were excluded. To obtain healthy control participants, we posted a recruitment notice on a website and selected 30 healthy sex‐ and age‐matched participants. These healthy volunteers had no history of bipolar disorder, schizophrenia, or other psychiatric illnesses and did not show any significant mood or thought symptoms as assessed via the MINI (Table 1). All participants were right‐handed as indicated by Annett's Handedness Questionnaire (Annett, 1970). Participants received W-----35,000 (approximately $35) for participating in the study and W-----10,000 (approximately $10) for performing the UG, regardless of their performance on the task. Written informed consent was obtained from all participants, and all participants displayed adequate understanding of the study procedures. This study was approved by the Institutional Review Board of Severance Mental Health Hospital and was conducted in accordance with the Declaration of Helsinki.

**Table 1:** Demogrphic and clinical characteristics

	Manic patients (*N* = 24)	Euthymic patients (*N* = 20)	Healthy controls (*N* = 30)	Stats (*F*, *t*, or *χ* ^2^)	*P* value
Age	32.8 ± 9.18	36.7 ± 10.1	35.9 ± 6.65	1.29	0.28
Sex (M/F)	13/11	12/8	17/13	0.15	0.93
Education (years)	13.6 ± 0.82	14.6 ± 1.96	13.7 ± 2.01		
Age at onset (years)	25.7 ± 8.46	26.7 ± 10.48		−0.36	0.72
Illness duration (years)	4.42 ± 2.02	4.75 ± 2.38		−0.50	0.62
Number of episode	2.58 ± 1.10	2.25 ± 1.12		0.99	0.33
YMRS	16.5 ±7.33	1.50 ± 1.73		9.73	<0.001
MADRS	4.92 ± 3.88	2.70 ± 2.05		2.42	0.02
GAF	51.1 ± 11.6	79.0 ± 16.7		−6.49	<0.001
IQ	106 ± 13.8	108 ± 13.4	111 ± 13.7	1.12	0.33
Lithium/Divalproex (N)	9/13	9/11		0.25	0.76
Chlorpromazine equivalent dose (mg)	797 ± 107	642 ± 112		4.67	<0.001

*Note*: YMRS, Young's Mania Rating Scale; MADRS, Montgomery‐ Åsberg Depression Rating Scale; GAF, The Global Assessment of Functioning; IQ, Intelligence Quotient.

### 2.2 Clinical assessment

We interviewed participants to assess their demographics, including age, sex, and educational level. We estimated clinical status using the Young's Mania Rating Scale (YMRS) (Young et al., 1978), the Montgomery‐Åsberg Depression Rating Scale (MADRS)(Åsberg et al., 1978), and the Korean version of the Global Assessment of Functioning (GAF) (Yi et al., 2003).

### 2.3 The ultimatum game

All participants played the role of the responder, not the proposer. They were told that the proposer was in another room. Each participant believed that they interacted with the proposer over a computer network. The responder was informed of the rules of the UG, that is, that they had to accept or refuse various offers. They were told that every decision to accept or refuse an offer would influence how much money the proposer would receive (Güth et al., 1982). Proposals did not change throughout the UG, regardless of the responders’ decisions. After the experiment, participants were debriefed that they played against the computer.

**FIGURE 1:**
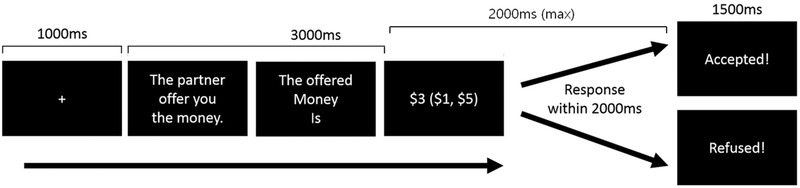
Schematic diagram of the ultimatum game for electroencephalography

The participants received a randomized series of 150 offers (50 trials for each of three conditions: 9:1, 7:3, 5:5). Participants were first presented with a fixation cross for 1000 ms, after which the proposer's offer appeared on the screen for 3000 ms. Following the offer presentation, the participant was instructed to press the keypad button to enter their response: The left button to accept the offer and the right button to refuse the offer. Once a response was recorded, the participant was shown a text message on the screen (“Accepted" or “Refused”) indicating their choice for 1500 ms. After a variable inter‐stimulus interval, a new trial was presented. Participants received a break between the initial assessment and completing the session (Figure 1). Stimulus presentation was done by using STIM^2^ software.

### 2.4 Electroencephalography recording

Electroencephalography (EEG) was recorded using a 64‐channel Neuroscan system (SynAmpsII) with a scalp AgCl lead cap. Electrodes were positioned according to the international 10/10 system. Two electrooculographic (EOG) electrodes were placed near the outer canthus and beneath the left eye to record eye movement. The recordings were referenced to linked electrodes placed on the left and right mastoid processes. To ensure stable skin conductance, all electrode impedances were maintained below 5 Ω. We recorded EEG data with a 0.01–200 Hz band‐pass filter at a sampling rate of 1000 Hz. The ground electrode was place3 on the forehead. Recordings were performed in a dimly lit, quiet, and electrically shielded room. Participants were seated in a comfortable reclining chair at an eye distance of 50 cm from the computer monitor (visual angle of 9º × 12º). Participants were instructed to concentrate on the center of the monitor and to avoid blinking to the greatest extent possible. Each participant's performance was monitored by a closed‐circuit camera. All participants remained awake for the duration of the procedure.

EEG analysis was performed off‐line. We used SCAN 4.3 software from Compumedics. Spurious EEG noises were removed by inspection. Spurious EEG noises were removed by inspection. The EEG signal was filtered using a 0.1–30 Hz band‐pass filter. To control for eye‐movement artifact, trials were adjusted via regression using the EOG (Semlitsch et al., 1986); artifacts were rejected if their amplitude exceeded ±100 μV. A low‐pass filter (8.5 Hz) was used to remove muscular movement, noise, and alpha‐wave activity. Artifact detection resulted in rejection of 1.9% of the segments. Additional eye movement detection resulted in rejection of a further 15.7% of the segments. EEG epochs of 800 ms were extracted. These 800 ms epochs consisted of a 200 ms baseline (i.e., the time before the onset of the offer) and 600 ms after the onset of the offer. ERPs were averaged between 200 ms before the stimulus onset and 600 ms after the stimulus onset.

### 2.5 Statistical analysis

Behavioral data and FRN amplitude as measured at the F_z_ and FC_z_ electrodes were analyzed using mixed‐model analysis of variance (ANOVA) to assess the effects of fairness (9:1, 7:3, and 5:5) and group (manic patients, euthymic patients, and healthy controls) on responses. Greenhouse‐Geisser correction for non‐sphericity was applied to all analyses. Pairwise comparisons to assess fairness in each block were performed following one‐way ANOVA; Bonferroni correction was applied to adjust for multiple comparisons. Pearson's correlation was conducted to assess the association between FRN amplitude and symptom severity. SPSS version 17.0 (SPSS Inc.; Chicago, IL USA) was used for all statistical analyses.

## 3 RESULTS

### 3.1 Rejection rates

**FIGURE 2:**
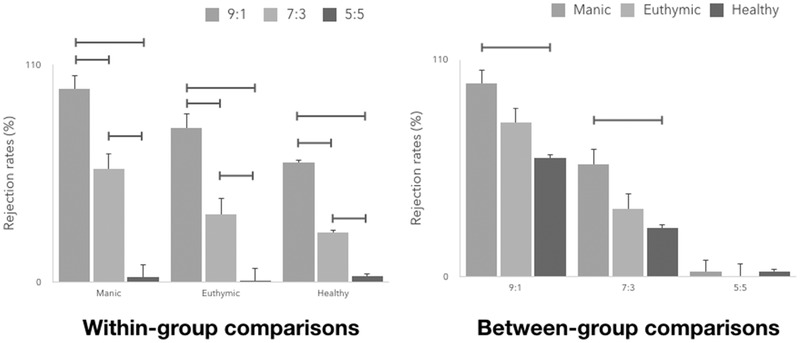
Rejection rates for within‐group comparisons and between‐group comparisons. Horizontal bars denote significant post hoc tests; Error bars denote standard errors

ANOVA revealed a significant main effect of fairness (F(2,142) = 194, *p* < 0.001) on rejection rates. There also was a main effect of Group (F(2,71) = 7.75, *p* = 0.001) as well as a fairness × group interaction (F(4,142) = 5.19, *p* = 0.001). We further explored this interaction in two ways. First, we assessed fairness within each group separately. Second, we assessed group effects separately for each level of fairness (Figure 2).

Within‐group analyses revealed significantly greater rejection rates to 9:1 offers compared to 7:3 or 5:5 offers in manic patients (F(2,46) = 18.2, *p* < 0.001; 9:1 offers vs. 7:3 offers, *p* < 0.001; 9:1 offers vs. 5:5 offers, *p* < 0.001; 7:3 offers vs. 5:5 offers, *p* < 0.001; rejection rates of 97.9 ± 7.21% for 9:1 offers, 57.3 ± 32.1% for 7:3 offers, and 2.71 ± 8.47% for 5:5 offers) and in euthymic patients (F(2,38) = 43.4, *p* < 0.001; 9:1 offers vs. 7:3 offers, *p* < 0.001; 9:1 offers vs. 5:5 offers, *p* < 0.001; 7:3 offers vs. 5:5 offers, *p* < 0.001; rejection rates of 78.1 ± 37.6% for 9:1 offers, 34.5 ± 38.9% for 7:3 offers, and 0.50 ± 0.89% for 5:5 offers) Similar results were observed among healthy controls (F(2,58) = 39.3, *p* < 0.001; 9:1 offers vs. 7:3 offers, *p* < 0.001; 9:1 offers vs. 5:5 offers, *p* < 0.001; 7:3 offers vs. 5:5 offers, *p* = 0.001; rejection rates of 60.4 ± 43.4% for 9:1 offers, 24.9 ± 36.3% for 7:3 offers, and 2.83 ± 9.62% for 5:5 offers).

ANOVA revealed that rejection rates to 9:1 offers were significantly higher in manic patients, but not in euthymic patients, compared with healthy controls (F(2,73) = 8.07, *p* = 0.001; manic vs. euthymic, *p* = 0.18; manic vs. control, *p* < 0.001; euthymic vs. control, *p* = 0.23). Similar findings were observed in response to 7:3 offers (F(2,73) = 5.60, *p* = 0.01; manic vs. euthymic, *p* = 0.12; manic vs. control, *p* = 0.004; euthymic vs. control, *p* = 0.99). Rejection rates were not different among the three groups in response to 5:5 offers (F(2,73) = 0.062, *p* = 0.54).

There were no differences in reaction times to all offers among the three groups (F(2.73) = 0.38, *p* = 0.69, 1.78 ± 0.82 s in manic patients, 1.66 ± 0.93 s in euthymic patients, 1.88 ± 0.85 s in heathy controls for 9:1 offers; F(2.73) = 1.66, *p* = 0.20, 2.16 ± 0.93 s in manic patients, 2.01 ± 1.01 s in euthymic patients, 1.70 ± 0.92 s in heathy controls for 7:3 offers; F(2.73) = 0.10, *p* = 0.91, 1.59 ± 0.80 s in manic patients, 1.49 ± 0.89 s in euthymic patients, 1.51 ± 0.88 s in heathy controls for 5:5 offers)

### 3.2 Feedback‐related negativity amplitude

**FIGURE 3:**
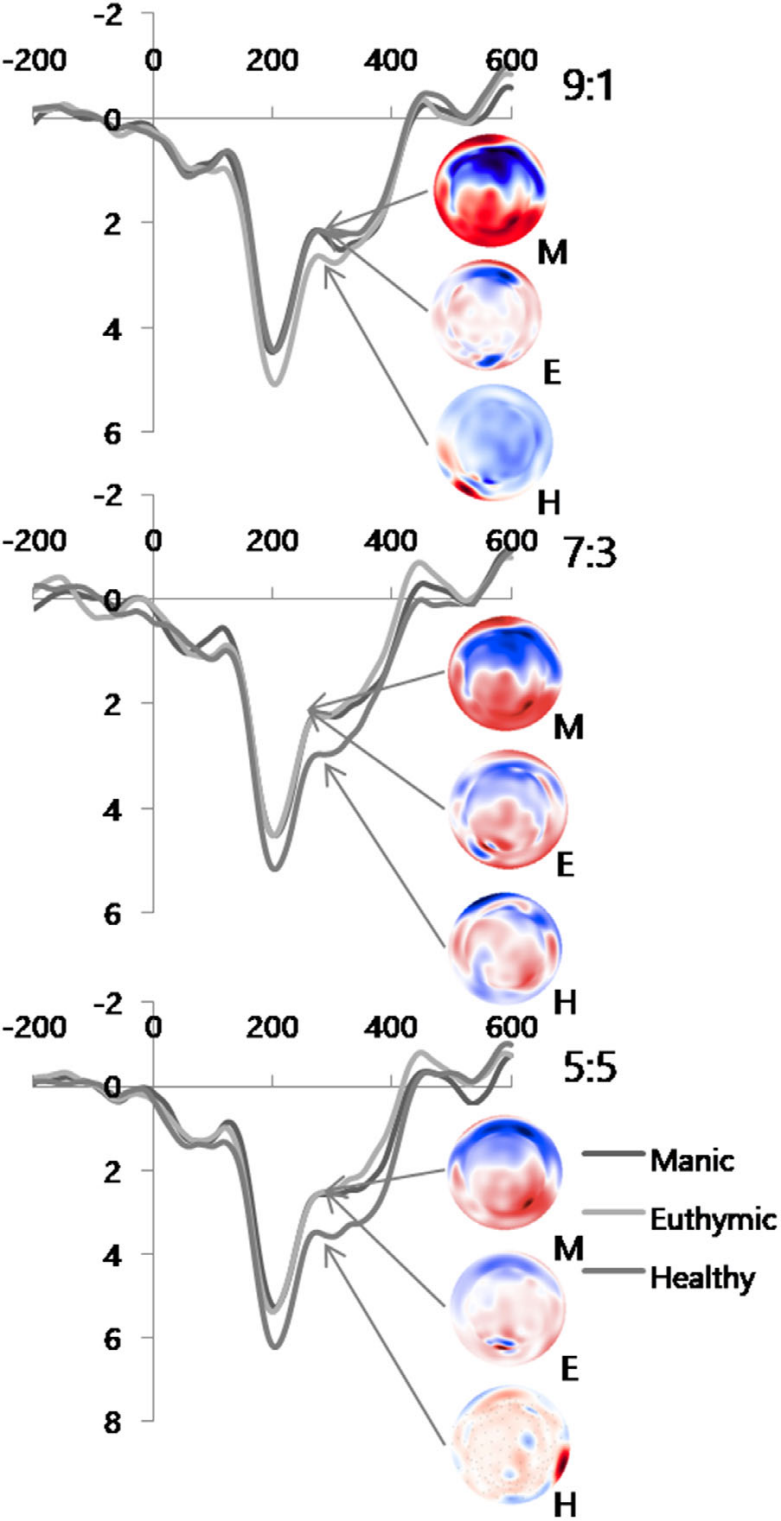
Grand average of feedback‐related negativity for three different offers in manic and euthymic patients and healthy controls

**FIGURE 4:**
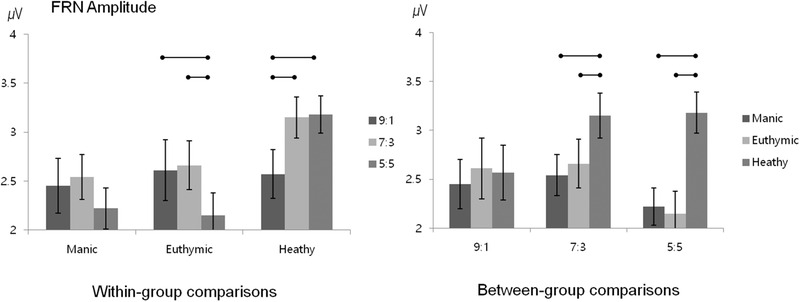
Feedback‐related negativity amplitudes for within‐group comparisons and between‐group comparisons. Horizontal bars denote significant post hoc tests; Error bars denote standard errors

FRN grand‐averages at the F_z_ electrode are shown in Figure 3. FRN amplitudes at F_z_ and FC_z_ electrodes were collapsed for analysis. A two‐way fairness × group ANOVA revealed a significant main effect of fairness (F(2,142) = 17.0, *p* < 0.001) and a significant fairness × group interaction (F(4,142) = 23.1, *p* < 0.001), but no main effect of group (F(2,71) = 1.87, *p* = 0.16). We further explored this interaction in two ways as described above for rejection rate (Figure 4).

Within‐group analysis revealed no significant difference in FRN amplitude for 9:1, 7:3, or 5:5 offers in manic patients (F(2,46) = 0.46, *p* = 0.63; FRN amplitudes: 2.45 ± 1.23 μV for 9:1 offers, 2.54 ± 1.25 μV for 7:3 offers, and 3.15 ± 1.16 μV for 5:5 offers). Euthymic patients exhibited greater (i.e., less positive) FRN amplitudes in response to 9:1 offers and 7:3 offers, compared with 5:5 offers (F(2,38) = 30.4, *p* < 0.001; 9:1 offers vs. 7:3 offers, *p* = 0.53; 9:1 offers vs. 5:5 offers, *p* < 0.001; 7:3 offers vs. 5:5 offers, *p* < 0.001; FRN amplitudes: 2.61 ± 1.23 μV for 9:1 offers, 2.66 ± 0.93 μV for 7:3 offers, and 2.15 ± 1.17 μV for 5:5 offers). Healthy controls exhibited greater FRN amplitudes in response to 9:1 offers, compared with 7:3 and 5:5 offers (F(2,58) = 23.4, *p* < 0.001; 9:1 offers vs. 7:3 offers, *p* < 0.001; 9:1 offers vs. 5:5 offers, *p* < 0.001; 7:3 offers vs. 5:5 offers, *p* = 0.63; FRN amplitudes: 2.57 ± 1.59 μV for 9:1 offers, 3.15 ± 1.16 μV for 7:3 offers, and 3.18 ± 1.00 μV for 5:5 offers). The main effect of fairness and interaction of fairness and group revealed that FRN responses to fair offers were different in healthy subjects with decreased negativity.

Between‐group comparisons of FRN amplitudes for each offer were also performed. ANOVA revealed that FRN amplitudes were significantly greater in manic and euthymic patients than in healthy controls in response to 5:5 offers (F (2,73) = 8.22, *p* = 0.001; manic vs. euthymic, *p* = 0.99; manic vs. control, *p* = 0.004, euthymic vs. controls, *p* = 0.003). FRN amplitudes did not differ across the three groups in response to 9:1 offers (F(2,73) = 0.83, *p* = 0.92) and to 7:3 offers (F(2,73) = 2.21, *p* = 0.12). Between group comparisons revealed that FRN response showed decreased negativity for 5:5 offers relative to manic and euthymic patients.

### 3.3 Correlations among clinical symptoms, rejection rates, and feedback‐related negativity amplitudes

Rejection rates and FRN amplitude for the three different offers were not significantly correlated with YMRS scores (*p* = 0.41 ∼ 0.95), MADRS scores (*p* = 0.13 ∼ 0.94), or GAF scores (*p* = 0.16 ∼ 0.92) in manic patients or in euthymic patients (YMRS scores, *p* = 0.37 ∼ 0.89; MADRS scores, *p* = 0.56 ∼ 0.92; GAF scores, *p* = 0.18 ∼ 0.68).

## 4 DISCUSSION

In this study, we investigated behavioral and electrophysiological characteristics related to social decision‐making and fairness among manic and euthymic bipolar patients compared to healthy controls, using the UG. All three groups displayed standard behavior with respect to rejecting unfair offers and accepting fair offers. Manic patients, but not euthymic patients, however, rejected unfair offers at a significantly higher rate than healthy controls, suggesting that manic patients are sensitive to unfair offers and respond to abnormal bargaining behavior. Healthy controls exhibited significantly greater (i.e., more negative) FRN amplitudes in response to overtly unfair offers, than relatively fair offers. In contrast, euthymic patients with bipolar disorder exhibited greater amplitudes in response to fair offers than unfair offers, whereas manic patients exhibited no difference in FRN amplitudes across the three offer types. Our findings of a more pronounced FRN amplitude in response to unfair offers, compared with those of fair offers, in healthy controls are consistent with previous studies. This typical difference in FRN amplitude between unfair and fair offers was not observed in manic or euthymic patients with bipolar disorder.

Manic patients exhibit poor behavioral control and severe emotional dysregulation (Perry et al., 2009) along with maladaptive antisocial behavior and exploit others’ weaknesses, which may be associated with impaired social cognition (Marsh & Blair, 2008). Patients with bipolar disorder are more likely to have different views on moral judgment when faced with emotionally salient moral dilemmas, especially during the manic phase, than healthy individuals (Kim et al., 2015). Previous study reported the euthymic patients made angry expression about game and showed greater rejections for ambiguous unfair offers (Duek et al., 2014). Euthymic patients showed more reciproicity than healthy group, which is dysfunctional due to reduced their gains in the Trust game (Ong et al., 2017). Moreover, several studies have shown that rejection behaviors among healthy individuals result from emotional reactions to unfair offers in the UG (Chapman et al., 2009; Tabibn, 2008; Van't Wout et al., 2006 ). The rejection of unfair offers in the UG was associated with skin conductance activity, which can only be observed in humans not computers as proposers. This provides physiological support for economic models of emotional decision‐making in humans (Van't Wout et al., 2006). Our finding is consistent with previous studies in more rejection rates for ambiguous unfair offers in the manic patients, not in the euthymic patients. Therefore, impaired emotional regulation and elevated anger with existing executive impairment (S. J. Dutra et al., 2014; Sunny J Dutra et al., 2016) may be related to higher rejection rates of unfair offers among manic patients with bipolar disorder.

In this study, we demonstrated no pronounced change in amplitude (i.e., not more negative) to unfair offers among manic and euthymic patients with bipolar disorder. FRN has been reported to be generated in the ACC (Gehring & Willoughby, 2002; Miltner et al., 1997) or medial prefrontal cortex (Becker et al., 2014; Carlson et al., 2011). The FRN elicited by the UG has been shown to originate in the ACC (Hewig et al., 2011). These brain regions play important roles in automatic and implicit emotional regulation and show decreased functional connectivity or activity in bipolar disorder (Etkin et al., 2015; Phillips & Swartz, 2014). A recent review (Chase & Phillips, 2016) suggested that functional interactions between the amygdala and medial prefrontal cortex are altered in bipolar disorder, and that this disconnectivity pattern may result in impaired amygdala regulation. The amygdala has been implied to be an important structure for emotion and decision‐making with physiological components (Bechara et al., 1999). These abnormalities in neural function may be associated with the observed changes in the rejection rate and altered FRN amplitude pattern observed among manic patients in the current study. Generally, the FRN has a relatively negative amplitude in response to outcomes that are worse than expected and a relatively positive amplitude in response to outcomes that are better than expected (Hajcak et al., 2005; Holroyd et al., 2008). The UG reflects an evaluation of decision‐making that is linked to social interactions. In this context, patients with bipolar disorder who exhibit a low FRN amplitude in response to unfair offers may have problems interpreting behavioral or verbal cues. Similarly, the FRN amplitude elicited by the UG is significantly less pronounced in patients with schizophrenia than healthy controls (Horat et al., 2018). Antisocial offenders also exhibited reduced amplitudes on the UG (Mayer et al., 2018). These data suggest that patients with serious psychiatric illnesses may have deficits in decision‐making in social contexts.

In the current study, although rejection rates were significantly high in manic patients only, FRN amplitudes elicited by unfair offers were not different across manic and euthymic patients, despite their different mood states. Our group has previously demonstrated that the FRN amplitude elicited by a probabilistic reward task is significantly altered in both manic and euthymic patients with bipolar disorder (Ryu et al., 2017). Our current findings suggest that there is no correlation between mood symptom severity and rejection rates or FRN amplitude, that behavioral responses to unfair offers may be mood state‐dependent whereas electrophysiological alteration may be a trait abnormality.

The game theory assumes that all participants act rationally and aim to maximize their self‐interest (Leonard, 1995). In terms of the game theory, the responder in the UG should accept any offer above zero because any positive offer will be better than receiving nothing. Under the assumption that the responder acts rationally, the most effective strategy for the proposer is to make a minimal offer. Prior evidence shows, however, that responders tend to resist unfair offers and, as such, do not behave rationally. Responders typically reject a large proportion of unfair offers to resist and punish the proposer. An individual's emotional state can alter social bargaining behavior (Hewig et al., 2011). A study of patients with major depression revealed that such individuals reject unfair offers more often than healthy controls because of an enhanced tendency toward altruistic punishment among individuals with depression(Scheele et al., 2013). As a proposer, individuals with depression tend to offer more money to avoid rejection; these individuals also exhibit low mentalizing ability (Destoop et al., 2012). Negative affect leads to increased FRN, whereas state happiness leads to increased acceptance of unfair offers (Riepl et al., 2016). The influence of affective state on bargaining responses to social fairness, therefore, may be the reason for the altered responses observed in manic patients in the current study.

Our study has some limitations. First, we did not control the psychotropic medications taken by the patients; however, reports suggest that ERPs are not affected by antipsychotic drugs (Ford et al., 1994). To our knowledge, however, no study has evaluated the effects of antipsychotics on FRN. In this study, we did not find any significant correlation between medications (in chlorpromazine‐equivalent doses) and FRN amplitude in our patient groups. We also did not find any significant difference in FRN amplitudes at the F_z_ electrode between patients taking lithium and those taking valproate. Lithium and valproate for treating bipolar patients may have varying effects on cognitive functioning and have been shown to be associated with mild cognitive impairment, although some benefits have also been reported (MacQueen & Young, 2003). Our sample size was not large enough to allow us to completely exclude the possibility of medication effects. Second, we did not evaluate individual preferences for fairness. If we had evaluated participants in the dictator game, which directly evaluates the participants’ altruistic behavior, we could have assessed the direct relationship between fairness preference and FRN amplitude. Future studies will need to elucidate the mechanisms underlying fairness preferences. Third, we did not test the participants’ alcohol and/or drug levels. We only checked the mental status and drug history by inspection. Finally, we did not directly measure social cognition and decision‐making. Therefore, we do not know the association between the rejection rate or ERP amplitude and social decision‐making.

## 5 CONCLUSION

The main advantage of our experiment was that it addressed the bargaining abilities and related neurophysiological changes in patients with bipolar disorder. Our observations suggest that aberrant interactive decision‐making behaviors could be a state marker for mood state‐dependent symptomatic abnormalities whereas electrophysiological alterations elicited by unfair offers may represent trait abnormalities irrespective of mood states. Taken together, these findings support the proposition that emotional decision‐making in bipolar patients is dysregulated regardless of mood status. Further research characterizing emotional decision‐making in patients with bipolar disorder may enable clinicians to understand the patients’ behavior.

## CONFLICT OF INTEREST

None

### PEER REVIEW HISTORY

The peer review history for this article is available at https://publons.com/publon/10.1002/brb3.2289

## PEER REVIEW HISTORY

The peer review history for this article is available at https://publons.com/publon/10.1002/brb3.2289

## Data Availability

The data that support the findings of this study are available from the corresponding author upon reasonable request.
